# Diagnosis and Management of Atrial Arrhythmias in Cancer Patients

**DOI:** 10.1007/s11886-025-02261-4

**Published:** 2025-06-25

**Authors:** Robert Monsour, Angie Seo, Nicholas Wilcox, Michael G. Fradley

**Affiliations:** 1 Department of Medicine, Perelman School of Medicine, University of Pennsylvania, Philadelphia, PA USA; 2 Thalheimer Center for Cardio-Oncology, Division of Cardiology, Perelman School of Medicine, University of Pennsylvania, Philadelphia, PA USA

**Keywords:** Atrial fibrillation, Cardio-oncology, Cardiotoxicity, Atrial arrhythmias

## Abstract

**Purpose of Review:**

Atrial arrhythmias are an increasingly recognized cardiovascular issue for cancer patients and can lead to increased morbidity and mortality independent of the oncologic prognosis. This review highlights the epidemiology of atrial arrhythmias in cancer patients including risk factors and arrhythmogenic cancer treatments as well as the current state of knowledge regarding the prevention and treatment of atrial arrhythmias in this population.

**Recent Findings:**

There appears to be bidirectional risk of atrial arrhythmias and cancer, with increased rates of atrial fibrillation identified in cancer patients but also increased cancer in patients with know atrial fibrillation. Moreover, as the cancer treatment landscape has continued to evolve, many novel treatments have been shown to be arrhythmogenic. Currently, the recommendations for the prevention and treatment of cancer associated arrhythmias are the same as the general population.

**Summary:**

Atrial arrhythmias are common in cancer patients due to multiple factors including shared risk factors and the cancer treatments themselves. Additional research is essential to better understand risk and also to provide cancer-specific recommendations for the prophylaxis and management of atrial arrhythmias and their associated thromboembolic complications.

## Introduction

The treatment landscape for cancer has dramatically evolved over the last several decades leading to increased survival rates for most malignancies. Despite these positive advancements, toxicities, particularly those impacting the cardiovascular system, can limit the successful delivery of these treatments. Arrhythmias are an increasingly recognized cardiovascular issue in cancer patients, driven both by the underlying malignancies and their treatments, and can have significant impact on patient morbidity and mortality. Atrial arrhythmias, including atrial fibrillation and flutter, are most commonly encountered rhythm disturbances in this populations and their management requires a careful and nuanced approach to minimize adverse effects and allow for the continued delivery of potentially life-saving cancer treatments. In this review, we discuss the epidemiology and mechanism of atrial arrhythmias in cancer patients and the current recommendations for their optimal management. In this endeavor, given their significant overlap in risk factors and management strategies, our discussion will treat atrial fibrillation and atrial flutter as similar phenomena; presented data, however, will still reference which arrythmia was observed in each composite study.

## Association of Cancer and Atrial Arrhythmias

### Epidemiology

There is a bidirectional relationship between cancer and cardiovascular disease, including atrial fibrillation (AF), due to both shared risk factors and underlying pathophysiologic mechanisms [1, 2]. Several epidemiologic studies have shown an association between cancer and increased risk of AF [2–6]. In a study using Dutch nationwide statistics between 2015 and 2019, 472,745 participants diagnosed with incident AF had an increased one-year incidence of cancer (2.8%) versus non-cancer controls (1.2%) (adjusted Hazard ratio [aHR] 2.78, 95% CI 2.69, 2.87) [2]. In a study of 85,423 patients with breast cancer from 2007 to 2014 employing the Surveillance, Epidemiology, and End Results (SEER) Medicare registry, there was an increased cumulative incidence of AF relative to propensity-matched non-cancer controls over a 1-year follow-up period (aHR 1.98, 95% CI 1.73, 2.27) [3]. A study of 816,811 patients with cancer and over 1.6 million control subjects without cancer from the Korean National Health Insurance Service database from 2009 to 2016 found that cancer was an independent risk factor for incident AF (aHR 1.63, 95% CI 1.61, 1.66) [4]. Furthermore, the incidence of AF varied by cancer type. For example, multiple myeloma (aHR 3.34, 95% CI 2.98, 3.75) and lung cancer (aHR 2.69, 95% CI 2.45, 2.95) were associated with the highest risk of incident AF among hematologic and solid malignancies, respectively [4]. In a nationwide Danish cohort study of 310,000 individuals diagnosed with cancer between 2000 and 2012, there was an increased incidence of new-onset AF compared to non-cancer controls (adjusted incident rate ratio 1.45, 95% CI 1.44, 1.48), and this association was observed for 12 of 13 major cancer subtypes examined [5]. A meta-analysis of 5 observational studies comprising over 5.8 million subjects found that patients with solid tumors had an increased risk of developing AF compared to non-cancer controls (OR 1.47, 95% CI 1.31, 1.66, I^2^ = 0.67), and that the risk of AF was highest within 90 days of cancer diagnosis [6]. 

Reciprocally, AF has also been associated with an increased risk of cancer [2, 7, 8]. 320,139 participants in a study using Dutch nationwide statistics diagnosed with incident AF had an increased one-year incidence of cancer (2.5%) compared to non-cancer controls (1.8%) (aHR 1.52, 95% CI 1.46, 1.58). A Danish population-based prospective cohort study of 26,222 men and 28,879 women showed that incident AF was associated with an increased risk of any cancer type (HR 1.41, 95% CI 1.26, 1.58 for men, HR 1.15, 95% CI 1.02, 1.32 for women) [7]. This corroborated the findings of a prospective cohort study of 34,691 women followed for incident AF and cancer between 1993 and 2013, which showed that incident AF was associated with an increased risk of cancer (aHR 1.48, 95% CI 1.25, 1.75) [8]. The relative risk of cancer was highest in the first 3 months following initial AF diagnosis, but remained significant at 1 year [7, 8]. 

### Outcomes

AF and cancer may be associated with an increased risk of adverse clinical outcomes including bleeding and mortality [2, 3, 8–11]. In the ROCKET-AF and ENGAGE AF-TIMI 48 randomized clinical trials of anticoagulation in patients with AF, malignancy was associated with an increased bleeding and mortality risk, but not ischemic events [10, 11]. In the study using Dutch nationwide statistics, cancer diagnosed following incident AF (aHR 7.77, 95% CI 7.45, 8.11) and AF diagnosed after incident cancer (aHR 2.55, 95% CI 2.47, 2.63) were both associated with significantly increased all-cause mortality, and the strength of the association was observed to vary by cancer type [2]. Patients with breast cancer in the SEER-Medicare registry analysis who developed new-onset AF within 30-days of their cancer diagnosis had a significantly increased risk of both all-cause mortality (aHR 2.15, 95% CI 1.32, 3.48) and cardiovascular mortality at 1 year (aHR 3.00, 95% CI 1.28-7.00), but not breast cancer mortality (aHR 1.42, 95% CI 0.77, 2.62) [3]. A prospective cohort study of 34,691 women, which showed that new-onset AF trended towards increased mortality from any cancer but did not reach significance (aHR 1.32, 95% CI 0.98, 1.79). A retrospective cohort study of over 2.4 million adults hospitalized with AF followed for a median of 2 years showed that patients with cancer, relative to those without, had an increased risk of multiple adverse clinical outcomes. This included all-cause death (aHR 2.00, 95% CI 1.99, 2.01), major bleeding events (aHR 1.27, 95% CI 1.26, 1.28) and intracranial hemorrhage (aHR 1.07, 95% CI 1.05, 1.10), but not cardiovascular death (aHR 1.01, 95% CI 0.99, 1.02) or ischemic stroke (aHR 0.95, 95% CI 0.93, 0.97) [9]. Of note, thromboembolic and bleeding risk varied depending on cancer type. Moreover, the negative association between AF and ischemic stroke in patients with cancer was attributed to the competing risk of other clinical outcomes, including all-cause mortality [9]. 

### Mechanisms

The increased risk of AF in patients with cancer is multifactorial, due to treatment-related adverse effects from cancer therapy and surgery, as well as shared risk factors such as older age, hypertension and diabetes mellitus. However, epidemiologic studies suggest that cancer itself is an independent risk factor for AF [4, 5]. This may be due to common mechanisms between cancer and AF, such as chronic inflammation and clonal hematopoiesis of indeterminate potential (CHIP), which increase the risk of both conditions [12]. More recent data highlight the importance of this shared pathophysiology. Chronic inflammation leads to immune suppression, which promotes tumor progression, while also facilitating AF through NLRP3 inflammasome activation in atrial cardiomyocytes and via both direct cardiac and systemic effects of pro-inflammatory cytokines such as TNF-α, IL-1, IL-6 and IL-17 [13–15]. CHIP, the age-related expansion of hematopoietic stem cells with pre-leukemic mutations, is associated with an increased risk of both cancer and cardiovascular disease [16–18]. However, recent studies have shown that CHIP is associated with an increased risk for incident arrhythmias including AF, potentially mediated by dysfunctional calcium handling, NLRP3 inflammasome activation and IL-6 production, and potentially associated with worse clinical outcomes [19–21]. Future research into the mechanisms underlying atrial fibrillation in patients with cancer is necessary to improve our understanding of the bidirectional relationship between these two conditions and to inform the development of targeted therapeutic approaches.

### Cancer Treatments Associated with Atrial Arrhythmias

#### Alkylating Agents and Hematopoietic Stem Cell Transplantation

The use of hematopoietic stem cell transplantation (HSCT) has expanded to older adults with cardiovascular comorbidities and patients have often been exposed to various cardiotoxic agents [22]. As a result, atrial arrhythmias, heart failure, myocardial infarction, and pericardial effusion have all been reported with HSCT [22]. In fact, in a large observational study of 3354 adults who underwent autologous or allogeneic HSCT between 2008 and 2019, the most common cardiovascular complication within the first 100 days post-transplant was AF; 100-day incidence was 2.8% and 2.3% for autologous and allogeneic recipients, respectively and 5-year incidence was 6.7% and 6.9% for autologous and allogeneic recipients, respectively [23]. This may be related to the increased risk of infections, large fluid shifts, and electrolyte imbalances during HSCT, in addition to the use of arrhythmogenic alkylating agents like melphalan for conditioning therapy [22, 24]. In a database of 438 patients who received melphalan with bone marrow transplantation, 11% developed AF or SVT which was significantly higher than the 0.6–2% incidence rates of atrial arrhythmia seen with non-melphalan regimens [25]. The mechanism of melphalan-associated atrial arrhythmia is not known, but could potentially be from increased oxidative stress resulting in dysregulation of intra-cellular calcium cycling and cardiomyocyte contractile function [26]. 

#### Anthracyclines

Anthracyclines are widely used for the treatment of solid and hematologic malignancies, including Hodgkin lymphoma, non-Hodgkin lymphoma, acute leukemias, breast cancer, ovarian cancer, and sarcomas [27]. Despite their oncologic benefits, there is a dose-dependent risk of myocardial damage and subsequent cardiac dysfunction during treatment or up to several years after completion of treatment with anthracyclines [27, 28]. Non-specific ECG changes and a broad range of arrhythmias have also been observed within the first few hours of infusion [28, 29]. In one study reviewing 24-hour ambulatory heart rhythm monitors performed during the first and last cycles of anthracycline administration, supraventricular extrasystoles were the most commonly detected atrial arrhythmia and the incidence of AF was 10.3% [30]. AF has also been reported in up to 56.6% of patients with anthracycline-related cardiomyopathy, which is a high arrhythmia burden but not too different from that of non-cancer patients with ischemic heart disease or dilated cardiomyopathy [31]. Fortunately these events are rarely life-threatening and can be managed without modification in treatment regimen [28, 30]. 

#### Bruton Tyrosine Kinase Inhibitors

Ibrutinib is a first-generation Bruton tyrosine kinase (BTK) inhibitor that became standard of care for the treatment of many B-cell malignancies, including chronic lymphocytic leukemia and mantle cell lymphoma [32, 33]. With this discovery, however, > 4-fold increase in cardiac events, including AF, ventricular arrhythmias, heart failure, and hypertension were observed compared to those not on ibrutinib [32, 33]. In fact, the incidence of AF has been reported in up to 17% of patients taking ibrutinib [33–35]. This is thought to be from covalent, irreversible binding of ibrutinib to other kinases in the B-cell receptor signaling pathway because second-generation BTK inhibitors (e.g. acalabrutinib and zanubrutinib) with more selective targets have been associated with less adverse cardiac events [32–34]. The rates of AF were lower in several randomized controlled studies and meta-analyses comparing acalabrutinib with ibrutinib (9.4% versus 16%) and zanubrutinib with ibrutinib (2% versus 15%) [36–38]. A more selective, noncovalent, reversible BTK inhibitor pirtobrutinib was also developed for patients with B-cell malignancies refractory to first and second-generation BTK inhibitors, and AF rates remained low at 3.8% in a recent phase 1–2 clinical trial [39]. 

#### Cyclin-Dependent Kinase 4/6 Inhibitors

Cyclin-dependent kinases 4/6 (CDK4/6) inhibitors are often used in combination with endocrine therapy for adjuvant treatment of high-risk hormone receptor-positive metastatic and early-stage breast cancer [40]. Abemaciclib, palbociclib, and ribociclib are three CDK4/6 inhibitors approved by the US Food and Drug Administration that are overall well tolerated and safe from a cardiovascular standpoint but they have been associated with increased risk of hypertension, heart failure, and AF [40]. In a retrospective cohort study of patients in the OneFlorida Clinical Research Consortium, 4.9% of 1376 patients taking CDK4/6 inhibitors developed AF and there was no difference in incidence among the different CDK4/6 inhibitors [40]. Those who developed AF were generally older and with more cardiovascular comorbidities. The median time to event for AFL was 2.1 months and was associated with increased all-cause death at a 3-year follow-up with an aHR of 5.88 (95% CI, 3.56–9.73) [40]. QTc prolongation has also been observed in phase 3 clinical trials, mostly with ribociclib but its clinical implications, including the risk of torsades de pointes, and the mechanism by which CDK4/6 inhibitors have cardiotoxic effects are areas that warrant further investigation [40–43]. 

#### Immune Therapies

Immune checkpoint inhibitors (ICI) targeting various monoclonal antibodies like anti-CTLA-4, anti-LAG-3, anti-PD-1, and anti-PD-L1 have revolutionized the treatment of solid and hematologic malignancies including metastatic melanoma and triple-negative breast cancer [44]. 

The benefits of ICI come with high rates of immune-related adverse events of varying severity and of different organs [44]. Cardiovascular toxicities may manifest as myocarditis, non-inflammatory heart failure syndromes, myocardial infarction, conduction disease, or arrhythmias [44, 45]. The risk of atrial arrhythmias with ICI therapy is not well established, but there have been studies reporting AF rates of 2–30%, higher than that of myocarditis suggesting they may occur independent of myocardial inflammation [45, 46]. The role of PD-1 in the development of primary arrhythmias is an interesting area that warrants further investigation as there have been studies showing downregulation of PD-1/PD-L1 in AF patients compared to healthy controls and patients with persistent AF expressing lower levels of PD-1 than those with paroxysmal AF [44, 47]. Additionally, chimeric antigen receptor T-cell (CAR-T), bispecific T-cell engager (BiTE), and tumor infiltrating lymphocyte (TIL) therapies are cell-based immune therapies that have been successful in the treatment of various malignancies [44, 48]. While cardiotoxic effects of BiTE and TIL therapies are not well known, retrospective studies have shown cardiovascular complications in 10–20% of patients who developed high-grade cytokine release syndrome (CRS) after CAR-T therapy [48]. In addition to cardiomyopathy, heart failure, and myocardial infarction, various arrhythmias have been reported with an incidence rate of 5–12% [48]. In fact, arrhythmia was the most common cardiovascular event in the U.S. Food and Drug Administration Adverse Events Reporting System data [49]. It is unclear, however, if it is due to a direct effect of CAR-T therapy or a systemic inflammatory response seen with CRS [50–52]. 

#### Radiation Therapy

Radiation therapy (XRT) is often used in combination with chemotherapy and can increase the risk of accelerated coronary artery disease, valvular disease, pericardial disease, conduction disease, and heart failure when the heart is in the field of radiation [53]. In a retrospective cohort study of 233 patients, adverse cardiac events occurred in more than 20% of patients undergoing thoracic XRT for non-small cell lung cancer [54]. Atrial arrhythmias have commonly been reported with lung cancer XRT, with event rates as high as 17.1% [55]. In a retrospective analysis of 748 patients, AF was most common in patients with a history of coronary heart disease or prior arrhythmia, but atrial flutter and other SVT were not associated with baseline cardiovascular risk [55]. Radiation exposure to different cardiac substructures may also be predictors of cardiac events like these, as XRT to pulmonary veins was associated with increased risk for AF and SVT and XRT to the left circumflex coronary artery was associated with increased risk for atrial flutter in this same study [55]. In another retrospective cohort of 560 patients with lung cancer, the maximum dose of sinoatrial node was most predictive of AF and associated with increased mortality [56]. 

### Management of Atrial Arrythmias in Cancer Patients

#### Thromboembolic Prophylaxis

The prevention of thromboembolic events, particularly strokes is essential in patients with atrial arrhythmias. Current guidelines, validated in non-cancer patients, continue to recommend oral anticoagulation (OAC) for thromboembolism prophylaxis in patients with AF and a CHA_2_DS_2_-VASc score greater than or equal to 2 (for males) and greater than or equal to 3 (for females) with a HAS-BLED score less than 3 [57]. It is possible that this calculator underestimates the risk of thromboembolic events in cancer patients, who exist in a persistent pro-thrombotic state [58] and demonstrate a higher rate of strokes than the general population even without the presence of atrial arrythmias (adjusted SHR 4.15, 95% CI 3.29, 5.23) [59]. Despite this, many cancer patients may not be receiving guideline recommended anticoagulation– as high as 44.3% in one study [60]. Nevertheless, the competing risk of cancer-associated mortality may obscure the benefit of systemic anticoagulation in cancer patients with AF since the time to developing stroke or other systemic embolism is generally measured in years. Therefore, shared decision making and individualized treatment planning should occur before initiating systemic anticoagulation [61, 62]. 

The risk of systemic anticoagulation causing significant bleeding events is likewise not fully captured by the most common calculator in use, HAS-BLED [63]. This is especially true for hematologic malignancies as well as those involving the gastrointestinal or genitourinary lumens [64]. Moreover, many cancer patients have thrombocytopenia, however patients with platelet counts of less than 100,000 were excluded in each of the major direct oral anticoagulant trials for patients with atrial fibrillation [65, 66]. Furthermore, there exists minimal data regarding platelet thresholds for patients with active malignancy and concurrent atrial arrythmias. In one retrospective study of 61 patients with active hematologic malignancies and new atrial fibrillation with platelet counts less than 50,000, 30-day risk of clinically relevant bleeding was seen in 16.7% of patients on any anticoagulation versus 5.3% in patients who had anticoagulation held and restarted after platelet recovery, though this was not a statistically significant association (*p* = 0.3) [67]. Similarly, another retrospective study of 131 cancer patients with atrial fibrillation and platelet counts less than 50,000 found that the 30-day cumulative incidence of major bleeding was 7.8% and 2.44% in the group on anticoagulation versus not, respectively (HR 3.29 [95% CI 0.42–26.04]) [68]. As such, expert consensus is that below a platelet count of 50,000 it is reasonable to hold anticoagulation for atrial thromboembolic prophylaxis [69]. 

Even if there is no known history of cancer in a patient, bleeding on anticoagulation therapy can be the presenting symptom of a new cancer diagnosis [70]. This is supported by a population-based cohort study that found patients with AF who were started on warfarin or a DOAC and had a documented bleeding event had a higher risk of being diagnosed with cancer in the following two years (HR 4.0, 95% CI 3.8,4.3); bleeding site was even concordant with the location of the malignancy [70]. Cancers identified as a result of a bleeding event tended to be at an earlier stage (27.6% stage IV after bleeding vs. 31.3% without, *p* = 0.029)—further suggesting that an index bleeding event on anticoagulation for AF may warrant a neoplastic investigation rather than simply stopping therapy [70]. 

If the decision is made to prescribe anticoagulation for AF, current ACC/AHA/HRS guidelines recommend the use of DOACs [69]. This is supported by a several studies which show reduction in major bleeding events, venous thromboembolism (VTE) and strokes when compared to vitamin K antagonists (major bleeding events HR 0.73, 95% CI 0.56, 0.94,VTE HR 0.37, 95% CI 0.23, 0.61, ischemic stroke HR 0.59, 95% CI 0.46, 0.75) [71–73]. Additionally, both VKAs and DOACs are prone to drug-drug interactions (DDI) as they are metabolized by the CYP450 system and or P-glycoprotein glycoprotein transporters (P-gp) [74]. 

Another potential option for anticoagulation is low molecular weight heparin (LMWH). However, LMWH has never been validated for long-term anticoagulation for AF, but has rather been the preferred agent for VTE treatment in cancer patients—most likely due to its favorable pharmacokinetic profile [75]. Thus, it remains an off-label indication for a medication that can be logistically difficult to take with its twice daily subcutaneous dosing.

Left atrial appendage occlusion (LAAO) is an alternative to systemic anticoagulation for patients with nonvalvular AF from an irreversible cause with elevated bleeding risk [69]. Initial studies in patients with non-valvular AF, including PROTECT AF and PREVAIL, showed that the left atrial appendage occlusion device was noninferior to warfarin for the prevention of ischemic stroke and systemic embolic events [76, 77]. The device was found similarly to be noninferior to DOACs in studies such as PRAGUE-17 [78, 79]. The elevated bleeding risk and often persistent pro-thrombotic state of cancer patients make non-operative LAAO for primary thromboembolic prophylaxis an attractive option for this population. One prospective, single-center study evaluating percutaneous LAAO in 57 cancer patients (16.4% with metastatic disease, 25% actively undergoing treatment) and 332 without cancer found no difference in mortality (HR 1.3, 95% CI 0.72, 2.35), major bleeding events (HR 1.2, 95% CI 0.45, 3.33), and stroke (HR 0.64, 95% CI 0.19, 2.21) between the two groups at 3 years [80]. Another retrospective sub-analysis of patients with and without cancer from the Australian LAAC registry (11.7% with cancer) found no difference in 1-year survival (96.1% vs. 94.0%, *p* = 0.582) or 5-year event free survival (64.9% vs. 74.4%, *p* = 0.124) between the two groups; this was despite the cancer patient group being significantly older and with a greater proportion having had significant GI bleeds in the past [81]. Similar findings were obtained from 55 cancer patients undergoing LAAO at Mayo clinic sites when compared to patients without cancer; specifically, there was no significant difference in ischemic stroke (HR 0.44; 95% CI 0.10,1.97), bleeding complication (HR 0.71, 95% CI 0.28,1.86), or death (HR 1.39, 95% CI 0.73, 2.64) [82]. However, there is minimal data comparing safety and efficacy outcomes between DOACs and percutaneous LAAO in cancer patients with atrial arrythmias. Despite this, there are lower rates of LAA closure in cancer patients (aOR, 0.76, 95% CI 0.74, 0.78) [83] suggesting clinicians have additional considerations, such as VTE prophylaxis and frailty, when choosing between OAC and LAAO [83]. 

Rate and Rhythm Control.

For cancer patients, much of the standard recommendations for rate and rhythm control are applicable. For rate control in atrial arrythmias without heart failure, it is reasonable to target a heart rate of < 100 to < 110 beats per minute [69]. In patients with heart failure with reduced ejection fraction (HFrEF) the goal heart remains less than 80 beats per minute at rest and less than 110 with moderate exercise [69]. Various options exist to achieve rate control however DDI may limit their use in cancer patients. For example, non-dihydropyridine calcium channel blockers are CYP3A4 inhibitors [84] digoxin is a P-gp substrate [85] and certain beta blockers are affected by CYP2D6 metabolism [86]. It is essential to review the various treatments a patient is receiving before deciding on a rate controlling agent in order to ensure patient safety.

Rhythm control is an option for symptomatic patients or in those patients with complications from their arrhythmias such as heart failure [69]. More recent data from the EAST-AFNET 4 study suggests that early rhythm control, even in asymptomatic individuals, may be preferred, reporting lower composite rates of death from cardiovascular causes, stroke, or hospitalization with worsening of heart failure or acute coronary syndrome (HR 0.79, 96% CI 0.66, 0.94) [87]. 

Several studies have evaluated the efficacy of anti-arrhythmic drugs (AADs) in cancer patients. In a retrospective trial of cancer patients with AF/AFL, ibutilide was 75% effective in restoring sinus rhythm and there were no significant adverse events [88]. A RCT showed that prophylactic use of amiodarone was effective in maintaining sinus rhythm in patients undergoing resection of lung cancer (absolute risk reduction of 23%, number needed to treat 4.4 [95% CI 3.1, 7.8]), however patients with pre-existing atrial arrythmias were excluded and chemotherapeutic use was not documented [89]. Despite these data, significant DDI can limit the use of antiarrhythmic drugs in cancer patients [90]. 

For patients who remain symptomatic from their atrial arrythmia despite AAD initiation or in whom AADs are not tolerated/contraindicated, or who have HFrEF with intractable tachycardia or what is thought to be arrhythmogenic cardiomyopathy, catheter ablation for AF can be considered [69]. For cancer patients in the China-AF registry, catheter-based AF ablation resulted in similar rates of AF recurrence to non-cancer patients (43.8% vs. 51.1%, *p* = 0.88) and there was no difference thromboembolism, major bleeding, and mortality after adjusting for confounders [91]. Another retrospective analysis compared patients with a recent cancer diagnosis to those without cancer who underwent catheter ablation for AF; this study similarly found that freedom from AF at 12 months post-ablation did not differ between the two groups (83.3% vs. 72.5%, *p* = 0.28), nor did the rate of repeat ablation (20.7% vs. 27.5%, *p* = 0.29) [92].

However, retrospective analysis of the National Inpatient Sample database found that patients with cancer who underwent AF ablation had higher inpatient complication rates, driven by bleeding and infections (total complication rate 10.5% vs. 7.9%, *p* < 0.001, bleeding 3.2% vs. 1.8%. *p* = 0.002, infectious complications 2.1% vs. 0.8%, *p* < 0.001); there was no difference in periprocedural strokes or 30-day mortality [93]. This same study found that hematologic malignancies were associated with a higher rate of hemorrhagic complications (4.4% vs. 2.0%, *p* = 0.028).

## Conclusions

Atrial arrhythmias are a frequently seen in cancer patients. The increased rates can be attributed to shared risk factors and similar biological mechanisms linking the two diseases, as well as a toxicity of various cancer treatments. Despite these observations, there are often more questions than answers when it comes to managing these conditions. Recommendations for treating atrial arrhythmias in cancer patients mirrors that of the general population however it is increasingly clear that there are unique challenges that exist in this population which can impact optimal care delivery. It is essential for increased research and data in the field of arrhythmias and cardio-oncology in order to provide safe, effective and informed prevention and treatment of rhythm disorders in cancer patients.

## Key References

- Fradley MG, Beckie TM, Brown SA, et al. Recognition, Prevention, and Management of Arrhythmias and Autonomic Disorders in Cardio-Oncology: A Scientific Statement From the American Heart Association. *Circulation*. Jul 20 2021;144(3):e41-e55. doi:10.1161/cir.0000000000000986
-
  - Comprehensive statement/recommendations regarding the evaluation and management of arrhythmias in cancer patients.
- Yun JP, Choi EK, Han KD, et al. Risk of Atrial Fibrillation According to Cancer Type: A Nationwide Population-Based Study. *JACC CardioOncol*. Jun 2021;3(2):221–232. doi:10.1016/j.jaccao.2021.03.006
-
  - Comprehensive study demonstarting the epidemiology of atrial fibrillation and its association with various cancer types.
- Grewal K, Wang X, Austin PC, et al. Bleeding and New Malignancy Diagnoses After Anticoagulation for Atrial Fibrillation: A Population-Based Cohort Study. *Circulation*. Feb 20 2025;doi:10.1161/circulationaha.124.070865
-
  - Study showing bleeding events in anticoagulated patients with atrial fibrillation was strongly associated with a new cancer diagnosis highlighting a bidirectional risk between the two diseases.

## Data Availability

No datasets were generated or analysed during the current study.
